# Basic human values and the adoption of cryptocurrency

**DOI:** 10.3389/fpsyg.2024.1395674

**Published:** 2024-08-16

**Authors:** Adrian Stanciu, Melanie Partsch, Clemens M. Lechner

**Affiliations:** ^1^Faculty of Humanities, Education and Social Sciences (FHSE), University of Luxembourg, Luxembourg, Luxembourg; ^2^Department of Methodology and Statistics, Utrecht University, Utrecht, Netherlands; ^3^Survey Design and Methodology, GESIS-Leibniz Institute for the Social Sciences, Mannheim, Germany

**Keywords:** basic human values, cryptocurrency adoption, theory of planned behavior, inequalities, logit regression analysis

## Abstract

Cryptocurrency is an attempt to create an alternative to centralized financial systems using blockchain technology. However, our understanding of the psychological mechanisms that drive cryptocurrency adoption is limited. This study examines the role of basic human values in three stages of cryptocurrency adoption–awareness, intention to buy, and ownership–using the Theory of Planned Behavior (TPB). Logistic regression analysis was conducted on a quota sample of 714 German adults, and the results showed that openness-to-change values increased the likelihood of cryptocurrency awareness, while self-enhancement values increased the likelihood of intention to buy and ownership. These findings were consistent even after controlling for demographic characteristics, attitudinal beliefs, and perceived behavioral control, which are important factors in the TPB. The results suggest that basic human values may influence an individual’s decision to adopt cryptocurrency, but the transition from awareness to ownership may be influenced by socio-economic opportunities available to interested individuals.

## Introduction

Cryptocurrencies can be better understood as digital money that relies on cryptic algorithms for secure transactions (cryptocurrencies and digital money are here used interchangeably). Satoshi Nakamoto published in 2008 a white paper on what has become the most successful cryptocurrency so far—Bitcoin (Nakamoto, 2008). They argued that the current financial system can one day be decentralized and replaced with one that relies on a chain of mathematical algorithms (i.e., the blockchain technology). Financial transactions could be possible directly between involved parties (peer-to-peer transactions) while any intermediaries (e.g., banks) and fees are removed, such that people have direct control over their finances. What makes cryptocurrencies so appealing is that technological development has been leveraged to revolutionize the old concept of money. Cryptocurrencies have received a mixed reception thus far, not least as market indices showed high volatility and ultimately crashed in 2022 (for a review see Chohan, 2022).

In most countries, cryptocurrencies remain a fringe phenomenon attracting a small share of economically active adults. We still know too little about what motivates people to become involved with this form of money (e.g., Sudzina et al., 2021; Martin et al., 2022; Littrell et al., 2024). Is it that people see in cryptocurrencies an opportunity to make profit of or are they rather interested in the novelty it brings about? This research argues that the adoption of cryptocurrencies resonates with peoples’ values—motivational goals that inform their behaviors and evaluations of the world around them (Schwartz, 2012). We draw on the Theory of Planned Behavior (TPB; Ajzen, 1991; Bosnjak et al., 2020) and explore the role of values at levels of awareness, intention, and behavior in view of cryptocurrency adoption. Data for the study stem from a large heterogeneous online sample of German adults that was collected in February 2022.

### A psychology of cryptocurrency and its adopters

A psychology of cryptocurrency adopters is emergent with a few recurring observations. The use of cryptocurrencies, especially if it involves financial speculation, shares similarities with addictive behavior such as problematic gambling (Delfabbro et al., 2021a,b). Problematic gamblers perceive digital money as an income-generator much like other forms of speculative trading that require a constant monitoring of the stock market and use of sell/buy strategies to increase profit. Fear of missing out and anticipated regret that one might miss an opportunity to become financially well-off contribute to this addiction (Delfabbro et al., 2021a). Moreover, the adoption and use of digital currencies including cryptocurrencies is influenced by the Dark Tetrad personality traits. For instance, narcissistic traits are associated with positive attitudes toward cryptocurrencies, whereas Machiavellian traits are correlated with cryptocurrency buying intentions (Martin et al., 2022). Adopters of digital money seem to be less aggregable and more extrovert while they have a low self-control, and they tend to be male, young adults, and better educated (Sudzina et al., 2021; Steinmetz, 2022). Littrell et al. (2024) found that the strongest predictors of cryptocurrency ownership were being male, alternative news reliant, and anti-authoritarian ideologies adherent. A sense of community and trust in the cryptographic code coupled with a willingness for risk taking behavior further explain cryptocurrency ownership and intention to trade (Obreja, 2022).

We argue that a deeper discussion of the societal and environmental underpinnings of cryptocurrencies is key in further developing a psychology of cryptocurrency adopters. Cryptocurrencies rely on the ancient technology of cryptography for securing financial transactions directly between involved parties. They hold several similarities with other technological advancements from the recent past, for example the Internet and mobile phones. This form of finance is innovative and highly technologized, two core traits that are in tune with progressive rather than regressive societies. However, the introduction of digital money raises greater challenges.

As some have argued, cryptocurrencies such as Bitcoin are, in theory, a better financial system for combating economic inequalities when compared to gold as standard and fiat (current) money (Othman et al., 2020). This is because the cryptic system has inbuilt strategies for correcting for inflation, and it defines an environment where virtually everyone can coin new money (i.e., coin-mining). Nonetheless, it is unclear whether this revolution is going to close the gap between the poor and the rich, with some findings suggesting that it could even increase existent inequalities (Chohan, 2022).

More serious complications may derive from the system of digital money. For instance, it can be exploited to facilitate criminal activities (Kethineni and Cao, 2020). Among the associated illegal activities are money laundering, tax evasion, Ponzi schemes and kidnapping for ransom. Governments around the globe are racing to regulate the system (e.g., Solodan, 2019) but it remains to be seen whether this is possible, and whether it is a good thing after all.

Massive amounts of electric energy are needed for digital money to work properly, which raises questions of environmental sustainability (Gallersdörfer et al., 2020). For instance, the estimated energy consumption and carbon emissions for Bitcoin alone for 2019 were 45.8 TWh and 22.9 MtCO_2_, which roughly translated to the levels produced by nations of Jordan and Sri Lanka in a year (Stoll et al., 2019). Both the blockchain and the coin-mining technologies rely on a network of computers to solve highly complex mathematical puzzles that become incrementally complex and energy-demanding with increasing transactions as more coins become available on the market.

The challenges associated with a wide adoption of cryptocurrencies reflect issues that people face in general, namely, how to relate with others and the environment. To further develop a psychology of cryptocurrency adopters, we propose that involvement with this new form of finance resonates with value motivational goals in people, which precede behavior, theory suggests (Schwartz, 2012).

### Values

According to the Theory of Basic Human Values (TBHV; Schwartz, 2012; Schwartz et al., 2012), values are desirable goals that individuals pursue in coping with a finite set of existential needs regarding their biological nature, coordinated action and survival in groups. People across situations and societies must find ways to resolve these needs, which gives rise to a finite and universal set of values. The TBHV proposes at least 10 and at most 19 basic values that are organized in the human psyche in a circular-like structure according to goal-compatibility (see Supplementary Figure S1). For instance, universalism opposes power since the motivational goal of universalism is the protection of the welfare of all people, whereas the motivational goal of power is dominance over people and resources. Likewise, conformity conflicts with self-direction since the motivational goal of conformity is the restraint of impulses likely to violate social expectations or norms whereas the motivational goal of self-direction is independent thought.

The basic values can be aggregated to four higher-order values that reflect core motivational goals of people. These are openness-to-change vs. conservation and self-enhancement vs. self-transcendence. The former duality reflects tensions between the goal of challenging the status quo by pursuing stimulating activities that emphasize autonomy on the one hand and the goal of maintaining the status quo by pursuing traditional and secure activities on the other hand. The latter pair reflects conflicts between the goal of improving one’s personal wellbeing by pursuing power and focusing on own accomplishments on the one hand and the goal of improving the wellbeing of others by pursuing benevolent and altruistic activities and caring for the environment.

Value tensions as described in the circular structure drive and motivate human behavior (Schwartz, 2012). This means that people act in ways that reflect their motivational goals. For example, someone might decide to go on a year-long trip with just a backpack because of their strong commitment to values of stimulation and hedonism. Another person might choose to pursue a career in a high stress-reward environment because of their high preference for values of achievement and power.

Values first form during early socialization processes and continue to develop throughout the lifespan (Cieciuch et al., 2016; Smallenbroek et al., 2023). The preference for certain values can change throughout the lifespan, with life events and acute societal challenges contributing to people adjusting their motivational goals (for example due to the Covid pandemic; Daniel et al., 2022). Values can change due to changing circumstances for behavior (Fischer, 2017). For example, moving up (or down) the social ladder means that a person is confronted with a different set of needs than it was previously the case and therefore the person would have to adjust or reconsider their values.

### The present research

Rough estimates indicate that the percentage of individuals who owned cryptocurrency in 2021 across the globe surpassed 3.80%, with an anticipated upward trend in the years to come (Crypto.com, 2022). Additionally, central banks like the European Central Bank are exploring the possibility of supplementing fiat money with digital coins to remain relevant in our increasingly digital society (e.g., digital Euro; Brunnermeier and Landau, 2022). While we notice this staggering evolution of crypto-and digital currency adoption, we still have limited understanding of the psychological processes driving it. Specifically, it remains unclear what motivates people to adopt digital forms of money. This study investigates whether people adopt cryptocurrencies as a reflection of their value motivational goals.

The present research is conducted in Germany, a prototypical Western cultural context with high levels of self-determination and other orientation values (Witte et al., 2020) in addition to a very high standard of living (UNDP, 2024). A representative survey of German adult Internet users conducted in 2019 shows, for instance, that about 87% of the respondents had some knowledge of cryptocurrency and about 14% owned cryptocurrency (Blockchain Research Lab, 2020). Cryptocurrency owners belonged to the age group 35–49 (32%) followed by the age groups 25–34 (27%), 50+ (22%) and 18–24 (19%). Men owned by far more cryptocurrency than women (69% vs. 31%). Meanwhile, the cryptocurrency owners had at least a secondary degree (19%), but they were highly educated (held a university degree, 30%) overall. Research on human values show that men are more likely to purse achievement goals than women, while younger people are generally more drawn to explorative value motivational goals than older people and education appears to not make a significant difference in value pursuit in the long run (Schwartz et al., 2012; Smallenbroek et al., 2023), evidence that makes it conceivable to us that values are at least partly responsible for cryptocurrency ownership.

We use the TPB (Ajzen, 1991; Bosnjak et al., 2020) to explore the associations between values and adoption of cryptocurrency at various stages, including awareness, intention, and behavior. Notably, the TPB recognizes that values are one of the many possible background factors that can shape beliefs about the behavior in question (attitudes), about perceived social norms about the behavior (subjective norms), and about perceived control over performing the behavior (perceived behavioral control) (Fishbein and Ajzen, 2009). These beliefs ultimately determine an individual’s intention to perform a behavior, resulting in the behavior itself. For instance, if an individual believes that adopting cryptocurrencies is financially rewarding, that their social network is supportive of this behavior, and that they have the necessary skills to use cryptocurrencies effectively, they may hold positive attitudes toward this behavior, which may encourage them to adopt digital currencies.

Note that we use the TPB as a framework in choosing meaningful predictors of cryptocurrency adoption and do not test the process model empirically. Previous research has taken a similar approach, for instance, in the study of lifelong learning (Partsch and Landberg, 2024). Meanwhile, other studies have applied TPB directly, for example, in the study of determinants to religious tax behavior compliance (e.g., Bin-Nashwan et al., 2021).

We hypothesize that the potential for novelty and financial benefits presented by cryptocurrencies resonate with values of openness-to-change and self-enhancement, respectively. Specifically, the emergence of cryptocurrencies is attributed to the innovative application of the blockchain technology, which is slowly transforming the concept of money. This technology is perceived by some to offer an opportunity to create a decentralized financial system, thereby enabling people to achieve financial independence from traditional banking institutions. Individuals with a preference for motivational goals that involve trying new things and acting in ways that stimulate them may be motivated to adopt cryptocurrencies due to the novel restructuring of the financial system. Therefore, we propose that a value preference for openness-to-change will be associated with the adoption of cryptocurrencies. As such, we expect that people with a high preference for this value will be more likely to be aware of, intend to buy, and own cryptocurrencies (Hypothesis 1).

The emergence of Bitcoin and other cryptocurrencies has made it easier for people to engage in capital-building activities similar to stock-market trading through online or mobile platforms (Fang et al., 2022). This presents opportunities for individuals to build capital, which might be aligned with value motivational goals of seeking power and achieving financial success. Consequently, we propose that a preference for self-enhancement will be associated with the adoption of cryptocurrencies. Notably, we expect that people with a high preference for this value will be more likely to be aware of, intend to buy, and own cryptocurrencies (Hypothesis 2).

Furthermore, we explore cryptocurrency adoption considering the attitudinal and perceived behavioral control strands leading to behavior, as stated in the TPB. Individuals can develop attitudes toward cryptocurrencies once they are aware of them and can assess their level of understanding. Based on an OECD report in Asia (OECD, 2019), we identified attitudinal beliefs related to trading opportunities that cryptocurrencies offer, such as their suitability for capital investment, exchangeability for cash, and being the appropriate time for buying. We also identified attitudinal beliefs about perceived obstacles to a widespread adoption of cryptocurrencies, such as concerns about illegal transactions and government regulation, which may hinder the advancement of a decentralized monetary system. While we expect these beliefs to be associated with cryptocurrency adoption, we specifically hypothesize that individuals who believe in the potential of cryptocurrencies—they hold positive attitudes—will be more inclined to purchase and own them (Hypothesis 3). Conversely, we predict that individuals who hold more negative attitudes toward cryptocurrencies, including concerns about their risks and challenges, will be less likely to adopt them (Hypothesis 4). Finally, we hypothesize that people with a high level of understanding the concept of cryptocurrency will be more likely to adopt them (Hypothesis 5).

Both the TPB (e.g., Fishbein and Ajzen, 2009) and the value theory (e.g., Schwartz, 2012) highlight the importance of values in shaping human behavior, whether directly or through their influence on attitudes and beliefs about the target behavior. Thus, we posit that values are a key determinant of cryptocurrency adoption, and as such, we give them priority over attitudes and perceived behavioral control in our estimation models. Importantly, we expect that values will remain associated with cryptocurrency adoption even after controlling for attitudes and perceived behavioral control, as they capture more fundamental and enduring beliefs and motivations that guide behavior. We recognize that mediation analyses are not appropriate for our correlational data, and that experimental or longitudinal data would be needed to establish causal relationships between values, attitudes, perceived behavioral control, and cryptocurrency adoption (Bullock et al., 2010).

## Method

### Participants

*respondi AG* was commissioned for data collection in February 2022 (Bilendi and respondi since 2023). This firm operates in multiple countries in Europe including Germany and specializes in non-probabilistic panel research. Over 300.000 panelists are registered in Germany. The respondi AG firm was instructed to apply the present questionnaire in a sample using quotas for gender, education level, and age to match the register-based German census proportions from 2017. The firm delivered a complete dataset containing information from 794 participants, which was within the range (750–800) considered in an *a-priori* power analysis as sufficient in observing a small effect from a multiple regression. Note that the delivered data does not support inferences to the German population due to its non-probabilistic nature (Scherpenzeel and Bethlehem, 2011).

Eighty participants were removed from the analysis due to straightlining (i.e., providing identical responses on 15 consecutive items) or a lack of variation on value items (i.e., within-person standard deviation less than 0.50). The cleaned and final data for the present study came from *N* = 714 participants (age: *M* = 43.05, *SD* = 13.72; Min = 18, Max = 65). Of these, there were 52.94% women, 66% (self-)employed, and 31.70% had Abitur (i.e., higher education entrance qualification in the German educational system) (also see Table 1).

**Table 1:** Descriptive statistics and correlation coefficients between the study variables.

		*n*	M (*SD*)	1	2	3	4	5	6	7	8	9	10	11	12	13	14	15
Cryptocurrency adoption																		
1	Awareness	714	0.89 (0.31)	1														
2	Intention	636	0.17 (0.37)	…	1													
3	Behavior	637	0.15 (0.36)	…	0.51***	1												
Covariates																		
4	Age	714	43.05 (13.72)	0.00	−0.23***	−0.24***	1											
5	Female	714	0.53 (0.50)	−0.08*	−0.13***	−0.22***	0.02	1										
6	Abitur	714	0.32 (0.47)	0.14***	0.11**	0.12***	−0.18***	−0.02	1									
7	Employed	714	0.66 (0.47)	0.05	0.06	0.05	−0.06	−0.11***	0.11***	1								
Values																		
8	Self-enhancement	714	−0.93 (0.71)	−0.01	0.27***	0.33***	−0.42***	−0.18***	0.15***	0.06	1							
9	Openness to change	714	0.28 (0.47)	0.13***	0.07	−0.04	0.04	−0.02	0.09*	−0.02	−0.02	1						
Perceived behavioral control																		
10	Understands	637	2.38 (1.12)	…	0.35***	0.41***	−0.16***	−0.34***	0.18***	0.12***	0.21***	0.08	1					
Attitudes																		
11	Investment	426	3.40 (1.02)	…	0.02	−0.04	−0.09	0.09	0.01	0.00	0.01	−0.06	−0.03	1				
12	Money exchange	369	2.81 (1.12)	…	0.19***	0.29***	−0.04	−0.06	−0.07	−0.04	0.14**	−0.14**	0.15***	0.07	1			
13	State regulated	356	2.33 (1.12)	…	0.14**	0.27***	−0.17***	0.09	−0.11	−0.09	0.25***	−0.22***	0.00	0.10	0.37***	1		
14	Illegal commerce	423	3.87 (0.99)	…	−0.08	−0.11*	0.13**	−0.09	0.07	0.05	−0.22***	0.12*	−0.03	0.12*	−0.07	−0.17***	1	
15	Good time to buy	372	3.00 (1.11)	…	0.44***	0.35***	−0.27***	0.11*	0.05	0.00	0.29***	−0.03	0.18***	0.11*	0.36***	0.35***	−0.08	1

Demographic covariates were all dummy coded, except age. Means for dichotomous variables refer to percentages. … = Questions not asked for people who were not aware of cryptocurrencies. Correlations calculated based on pairwise available data. Scale anchors for attitudes were: 1—complete disagreement, 5—complete agreement. Scale anchors for values were: 1—not at all like me, 6—very much like me. ***p < 0.001, **p < 0.01, *p < 0.05.

### Measurement

Unless otherwise stated, all measurements were translated from English into German by a bilingual author. The German translation was checked by a second bilingual author who resolved remaining inconsistencies. The questions addressing cryptocurrency adoption, and the related attitudes and perceived behavioral control, were inspired by an OECD report on cryptocurrency adoption in Asia (OECD, 2019). Measurement for covariates was derived from the established instruments of the German General Social Survey (ALLBUS).^1^

#### Values

Human values were measured with the German version of the recently revised Portrait Value Questionnaire (PVQ-RR, Schwartz and Cieciuch, 2021). Fifty-seven brief value descriptions of a fictitious character were presented to the study participants. Men and women received their gender specific questionnaire. Study participants were asked to indicate how much they saw themselves similar to these fictitious characters on an asymmetric 6-points scale ranging from 1—*not at all like to me* to 6—*very much like me*.

We built from these items two axes that are well established and highly used in the literature, namely the higher-order values of openness-to-change and self-enhancement. All items were centered to individual values (person-centering), which has consequence for the way findings are interpreted: Person-centered values indicate the importance of one value relative to all others for each individual study participant. Eighteen items that measure values of achievement and power were aggregated to the higher-order dimension of self-enhancement (*α* = 0.85, 95% CI [0.84; 0.87]). Item examples for achievement and power are “It is important to him/her to have ambitions in life” and “It is important to him/her that people do what he/she says they would,” respectively. Nine items that measure values of self-direction and stimulation were aggregated to the higher-order dimension of openness-to-change (*α* = 0.80, 95% CI [0.78; 0.82]). Item examples for self-direction and stimulation are “It is important to him/her to make his/her own decisions about his/her life” and “It is important to him/her to take risks that make life exciting,” respectively.

#### Cryptocurrency adoption

##### Awareness

Study participants had to indicate which one of two possibilities was true in their case: Whether they have previously heard of digital-or cryptocurrencies or have never heard of them. Awareness was recorded as 1 (has previously heard) and 0 (has not heard). All participants had to answer the question.

##### Intention

Study participants had to choose one of three possible answers, namely whether they would like to own in the future digital-or cryptocurrencies, they would not like to own in the future digital-or cryptocurrencies, and currently uncertain about that. Intention was recorded as 1 (wants to own in the future) and 0 (does not want to own in the future or is uncertain about it). Only study participants who were aware of cryptocurrencies were asked this question.

##### Ownership

Study participants had to choose one of three possible answers once more, namely whether they currently owned digital-or cryptocurrencies, they previously owned digital-or cryptocurrencies, and had never owned any. Self-reported behavior was then recorded as 1 (owned digital-or cryptocurrency currently or in the past) and 0 (never owned). Only study participants who were aware of cryptocurrencies had to answer the question.

#### Attitudes

Attitudes related to three opportunities arising from cryptocurrency adoption were measured, namely that digital-or cryptocurrencies (1) are an investment opportunity rather than a means for payment and (2) can be easily exchanged for cash as well as (3) that the time is right for buying digital-or cryptocurrencies. Furthermore, attitudes related to two possible obstacles to a wide implementation of cryptocurrency adoption were also measured, namely that (1) digital-or cryptocurrencies facilitate criminal activities and (2) the state regulates digital-or cryptocurrencies. All items were measured on a 5-point Likert scale with anchors ranging from 1—*complete disagreement* to 5—*complete agreement*. Only participants who were aware of cryptocurrencies answered these questions.

#### Perceived behavioral control

Study participants were asked how well they understood digital-or cryptocurrencies. Answers were given on a 5-point Likert scale with anchors ranging from 1 = *not at all well* to 5 = *very well*. Only participants who were aware of cryptocurrencies had to answer the question.

#### Covariates

Age was self-reported in years. Gender was self-reported as male (0) or female (1). Finally, educational attainment was recorded as the accomplished highest level of schooling based on six ordinal categories representing the German school system and other. For the present study, we recorded as 1 (has Abitur) and 0 (all other categories: primary school with and without a diploma, secondary and tertiary school, university of applied science, and other certificate). The employment status was recorded using nine categories, which we recorded as 1 (employed or self-employed) and 0 (all other: unemployed looking and not looking for a job, household responsible, still study, trainee, pensioner, and other). These covariates were chosen because the reviewed literature suggests that cryptocurrency adopters tend to be young, male, highly educated, and with a financially well-off status.

### Analytical approach

The present analyses were carried out in R (R Core Team, 2018), with all study materials including scripts and data made open access at the associated OSF project: https://osf.io/n7qw4/?view_only=8a0175a9558a48fdae978fe922b9fc13 (blind review link; will be made publicly available upon publication).

Due to random missing observations in the attitudinal measures, only 284 cases had complete information. To address this issue, we employed the R package *mice* (van Buuren and Groothuis-Oudshoorn, 2011) to impute missing data using a multiple imputation approach (m = 100) that replaced the missing observation with a predictive mean matching algorithm (Rubin, 1986). The present results, including the marginal effects and the 95% confidence intervals, were obtained by combining information from the multiply imputed datasets. Additional information on the missing data imputation can be found in Supplementary Figures S2, S3.

Using strategically placed filters in the questionnaire, we created three levels of cryptocurrency adoption from our sample. To assess cryptocurrency awareness, all study participants were included. To examine the intention to purchase cryptocurrencies in the future, we filtered the sample to only include participants who were already aware of digital currencies before the study and had not previously owned any. Finally, to study cryptocurrency adoption, we further filtered the sample to only include participants who had prior knowledge of cryptocurrencies. For each of these levels of cryptocurrency adoption, we then estimated two nested models.

The first estimated model included the study covariates and values as predictors (model 1). This allowed us to gain insights relevant for hypotheses 1 and 2. The nested model added the attitudinal and perceived behavioral control measures (model 2). This allowed us to test hypotheses 3 to 5.

Note that the present dependent variables are all dichotomous thus we resolved to logit regressions for model estimation. Logit regressions calculate estimates on a scale of logarithmically transformed odds-ratio, which is cumbersome to interpret. We here report the average marginal effects (AME) with the associated 95% confidence intervals, and, for interpretation purposes, we plot the marginal effects (ME) of value preferences calculated at all other significant main effects in the estimated models. ME, also written as *dY/dX*, for dichotomous variables indicate how the probability of an outcome varies when a particular explanatory variable changes, considering all other variables in the model. AME are the average over the marginal effects calculated for all observations.

Model fit was evaluated against the Tjur’s *D* – the coefficient of determination, also known as one of the pseudo *R*^2^ for logit regressions (Tjur, 2009). Tjur’s *D* is similarly interpretable as the regular *R*^2^, where coefficients closer to 1 are evidence for the statistical explanatory power of the estimated model. Nested models were compared using the Likelihood Ratio (LR) test for multiply imputed data (Meng and Rubin, 1992), which is implemented in the R package *mice*. The LR tests the null hypothesis that the nested model equals the lower model. Should the probability be below 5 that the observed data is equal or more extreme under the null hypotheses (LR with an associated *p* < 0.05), then the upper model is preferred over the lower model.

## Results

### Descriptive statistics and profile of adopters

Descriptive statistics and correlations between the study variables are provided in Table 1. In the overall sample, approximately 89% of the study participants were aware of cryptocurrencies, while 17% had the intention to buy in the future and 15% had owned at some point cryptocurrency. These values are somewhat different once the strategically placed filters were considered. Among the participants who heard of cryptocurrencies but never held any prior to the study, approximately 8.5% said that they were considering buying some in the future. Notably, of the participants who were aware of cryptocurrencies, about 15.4% mentioned that they owned cryptocurrencies at the time of the study or had owned in the past (also see the Supplementary Figure S4).

Study participants who were aware of cryptocurrencies had an average age of 43 (*SD* = 13.67) and were mostly female (51%), employed or self-employed (67%) and had an Abitur (i.e., university entrance qualification) 1/3 of the times (34%). On average, they prioritized values of openness to change (*M* = 0.39, *SD* = 0.45) while they gave little priority to values of self-enhancement (*M* = −0.93, *SD* = 0.71).

The participants who were aware of cryptocurrencies, never held any but intended to buy some in the future were slightly younger, with an average age of 37 (*SD* = 11.96) and were female half of the time (50%), mostly employed or self-employed (65%) and had an Abitur 1/3 of the time (33%). On average, they highly prioritized values of openness to change (*M* = 0.37, *SD* = 0.51) and gave little priority to values of self-enhancement (*M* = −0.65, *SD* = 0.65).

Finally, the participants who were aware of cryptocurrencies and held at the time of the study or had held in the past cryptocurrencies were slightly younger, with an average age of 35 (*SD* = 10.53). They were female ¼ of the times (26%), predominantly employed or self-employed (72%), while they had an Abitur about half of the time (47%). They prioritized on average values of openness to change (*M* = 0.26, *SD* = 0.49) and offered low priority to values of self-enhancement (*M* = −0.38, *SD* = 0.59).

### Logit regressions

**Figure 1:**
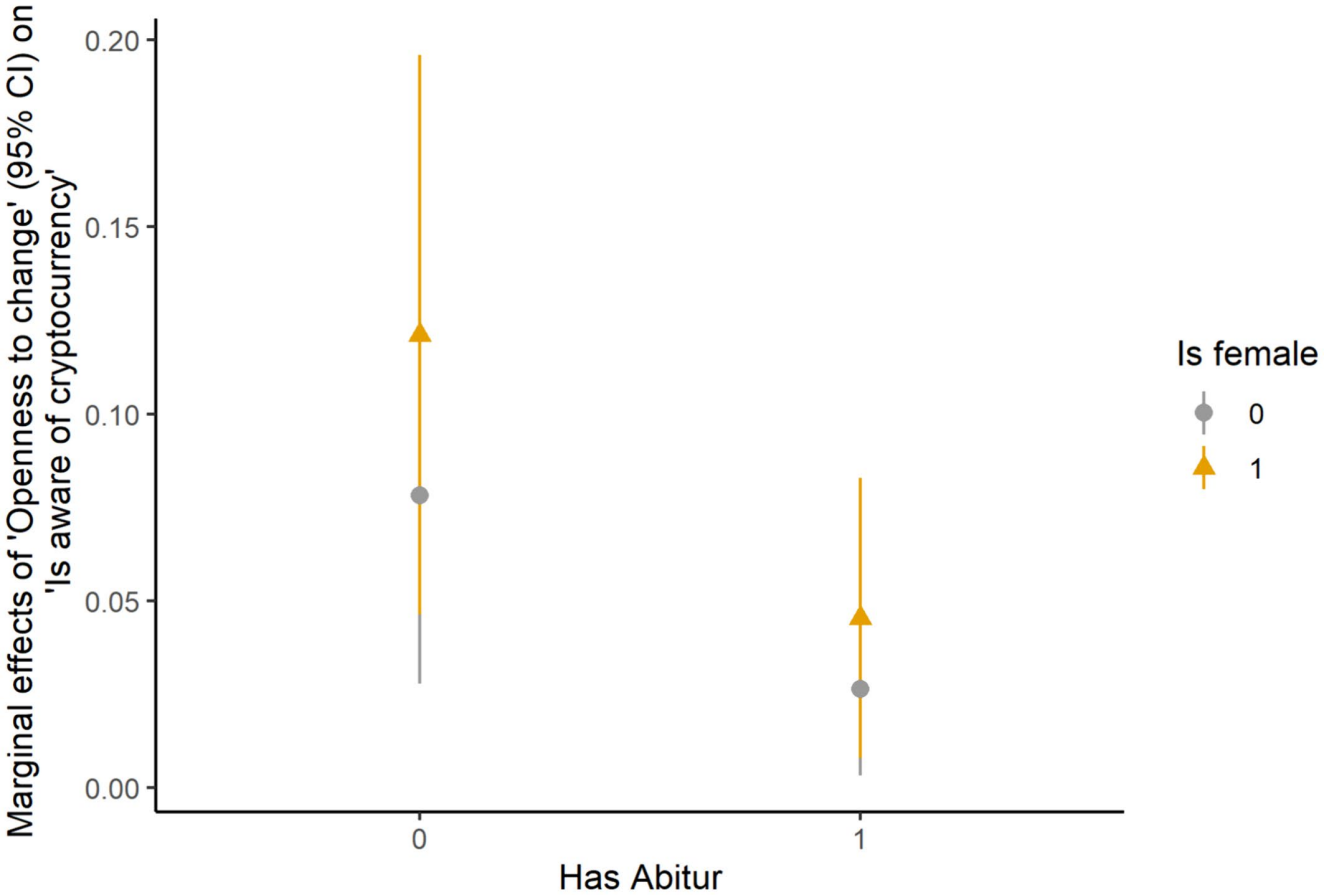
Marginal effects of openness to change on cryptocurrency awareness.

Results of logit regressions are shown in Table 2. We found support for hypotheses 1 and 2 at all levels of cryptocurrency adoption. According to estimations in model 1, which contained covariates and values, we found that values of openness-to-change were positively related to an awareness of cryptocurrency, but not with intention to buy in the future nor with ownership. On average, participants who prioritized values of openness-to-change were between 3 and 13 times more likely to be aware of cryptocurrencies. Notably, as can be seen in Figure 1, the ME of openness-to-change were higher among female participants compared to male participants, and, in general, higher for participants without an Abitur than those with one. This means that the likelihood of being aware of cryptocurrencies increases when prioritizing openness-to-change values, particularly for women and those without a higher education entrance qualification. Self-enhancement values, on the other hand, were not related to cryptocurrency awareness but were positively associated with the intention to buy and ownership. Participants who prioritized self-enhancement values were 1 to 9 times more likely to want to buy cryptocurrency in the future and 8 to 17 times more likely to hold cryptocurrency currently or previously.

**Table 2:** Average marginal effects for cryptocurrency awareness, intention, and behavior.

Predictor	Awareness							Intention							Behavior						
	*dY/dX*	*SE*	*95% CI*		*t*	*p*	*D*	*dY/dX*	*SE*	*95% CI*		*t*	*p*	*D*	*dY/dX*	*SE*	*95% CI*		*t*	*p*	*D*
			*LL*	*UL*						*LL*	*UL*						*LL*	*UL*			
Model 1							0.05							0.05							0.18
Covariates																					
Age	0.01	0.01	−0.01	0.01	0.34	0.73		−0.01*	0.01	−0.01	−0.01	2.52	0.01		−0.01**	0.01	−0.01	−0.01	3.06	0.01	
Female	−0.05*	0.02	−0.09	−0.01	2.30	0.02		−0.01	0.02	−0.06	0.03	0.65	0.51		−0.12***	0.03	−0.17	−0.07	4.44	<0.001	
Abitur	0.09***	0.02	0.05	0.13	4.31	<0.001		−0.02	0.02	−0.06	0.03	0.76	0.45		0.03	0.03	−0.02	0.09	1.28	0.20	
Employed	0.01	0.02	−0.03	0.06	0.24	0.62		−0.01	0.02	−0.05	0.05	0.08	0.93		0.01	0.03	−0.05	0.07	0.35	0.72	
Values																					
OCH	0.08**	0.03	0.03	0.13	3.11	0.01		0.03	0.02	−0.02	0.08	1.05	0.29		−0.02	0.03	−0.07	0.04	0.56	0.57	
SEN	−0.01	0.02	−0.05	0.02	0.85	0.39		0.05**	0.02	0.01	0.09	2.62	0.01		0.12***	0.02	0.08	0.17	5.50	<0.001	
Model 2 (No convergence)														0.15							0.40
Covariates																					
Age								−0.01	0.01	−0.01	0.01	1.91	0.05		−0.01**	0.01	−0.01	−0.01	2.87	0.01	
Female								−0.02	0.03	−0.07	0.03	0.86	0.39		−0.05*	0.02	−0.11	−0.01	2.15	0.03	
Abitur								−0.03	0.02	−0.07	0.02	1.16	0.25		0.01	0.02	−0.04	0.05	0.28	0.78	
Employed								0.01	0.02	−0.05	0.05	0.18	0.86		0.02	0.02	−0.03	0.07	0.77	0.44	
Values																					
OCH								0.02	0.02	−0.03	0.07	0.83	0.41		−0.01	0.03	−0.06	0.04	0.31	0.76	
SEN								0.05*	0.02	0.01	0.05	2.34	0.02		0.07***	0.02	0.03	0.10	3.57	<0.001	
Perceived behavioral control																					
Understands								0.03*	0.01	0.01	0.05	2.56	0.01		0.08***	0.01	0.06	0.10	7.01	<0.001	
Attitudes																					
Investment								0.01	0.01	−0.01	0.04	0.92	0.35		−0.02	0.01	−0.05	0.01	1.83	0.07	
Exchange								0.01	0.01	−0.02	0.03	0.36	0.72		0.02*	0.01	0.01	0.05	2.00	0.05	
Regulation								−0.02	0.01	−0.05	0.01	1.29	0.20		0.02	0.01	−0.01	0.05	1.60	0.13	
Illegal								−0.01	0.01	−0.03	0.01	0.95	0.34		−0.01	0.01	−0.03	0.02	0.65	0.65	
Good time								0.07**	0.02	0.03	0.11	3.89	0.01		0.05**	0.01	0.02	0.07	3.42	<0.001	

Awareness, 1 = has heard of cryptocurrencies, 0 = has never heard of cryptocurrencies. Intention, 1 = intends to own cryptocurrency in the future, 0 = does not intend to own cryptocurrency in the future or is unsure; Data was filtered for those who heard but never held cryptocurrencies. Behavior, 1 = owns cryptocurrencies now or has own in the past, 0 = has never owned cryptocurrencies. Abitur = basic education degree allowing university entrance, 1 = Abitur, 0 = all other basic education degrees. Employed = employment status, 1 = is employed, 0 = other statuses. OCH, Openness to change; SEN, Self-enhancement. Understands = How well understands cryptocurrencies. Investment = Cryptocurrencies are an investment opportunity and less a payment method, Exchange = Cryptocurrencies can be easily exchanged for cash, Regulation = The State regulates cryptocurrencies, Illegal = Cryptocurrencies facilitate illegal activities, Good time = Good time to buy cryptocurrencies. Step 2 for Awareness does not converge. Missing data for the five beliefs about cryptocurrency was imputed using a predictive mean matching algorithm, with results reported from pooled information over 100 imputations. dY/dX = average marginal effect. D = Tjur’s coefficient of determination that can be interpreted similarly to the regular R^2^. SE, standard error of estimate; LL, lower confidence bound; UL, upper confidence bound; *p*, probability level. *p < 0.05, **p < 0.01, ***p < 0.001.

The effects of self-enhancement values did not disappear in model 2, which added next to covariates and values also five attitudinal measures and one perceived behavioral control indicator. Model 2 was a substantial improvement over model 1, both in the case of predicting the intention to buy cryptocurrency in the future (ΔD = 0.10, LR = 6.19, *p* < 0.001) and ownership (ΔD = 0.22, LR = 15.76, *p* < 0.001). Model 2 for the prediction of cryptocurrency awareness did not converge and therefore the effects of openness-to-chance could not be further examined, which was anticipated since the study participants who answered that they never heard of cryptocurrencies did not receive further questions relating to cryptocurrency adoption.

We found support for hypothesis 3 and no support for hypothesis 4. One attitudinal belief that can be considered positive in view of cryptocurrency adoption–that it was a good time to buy cryptocurrency–was associated both with the intention to buy cryptocurrency in the future and ownership. On average, participants who held this belief were between 3 and 11 times more likely to want to buy cryptocurrency in the future, and between 2 and 7 times more likely to own cryptocurrency at the time of study or at some point in the past. One further attitudinal belief that can be considered positive–that cryptocurrencies can be easily exchanged for money–was associated with ownership. On average, participants who held this belief were between 1 and 5 times more likely to own cryptocurrency at the time of study or at some point in the past. None of the attitudinal beliefs that can be considered negative in view of cryptocurrency adoption were associated with intention to buy or ownership.

Results also provide evidence for our hypothesis 5, showing that perceived behavioral control–how well people understood cryptocurrency–was associated both with the intention to buy cryptocurrency in the future and ownership. On average, people who understood well cryptocurrency were between 1 and 5 times more likely to want to buy cryptocurrency in the future, and between 6 and 10 times more likely to own cryptocurrency at the time of study or at some point in the past.

**Figure 2:**
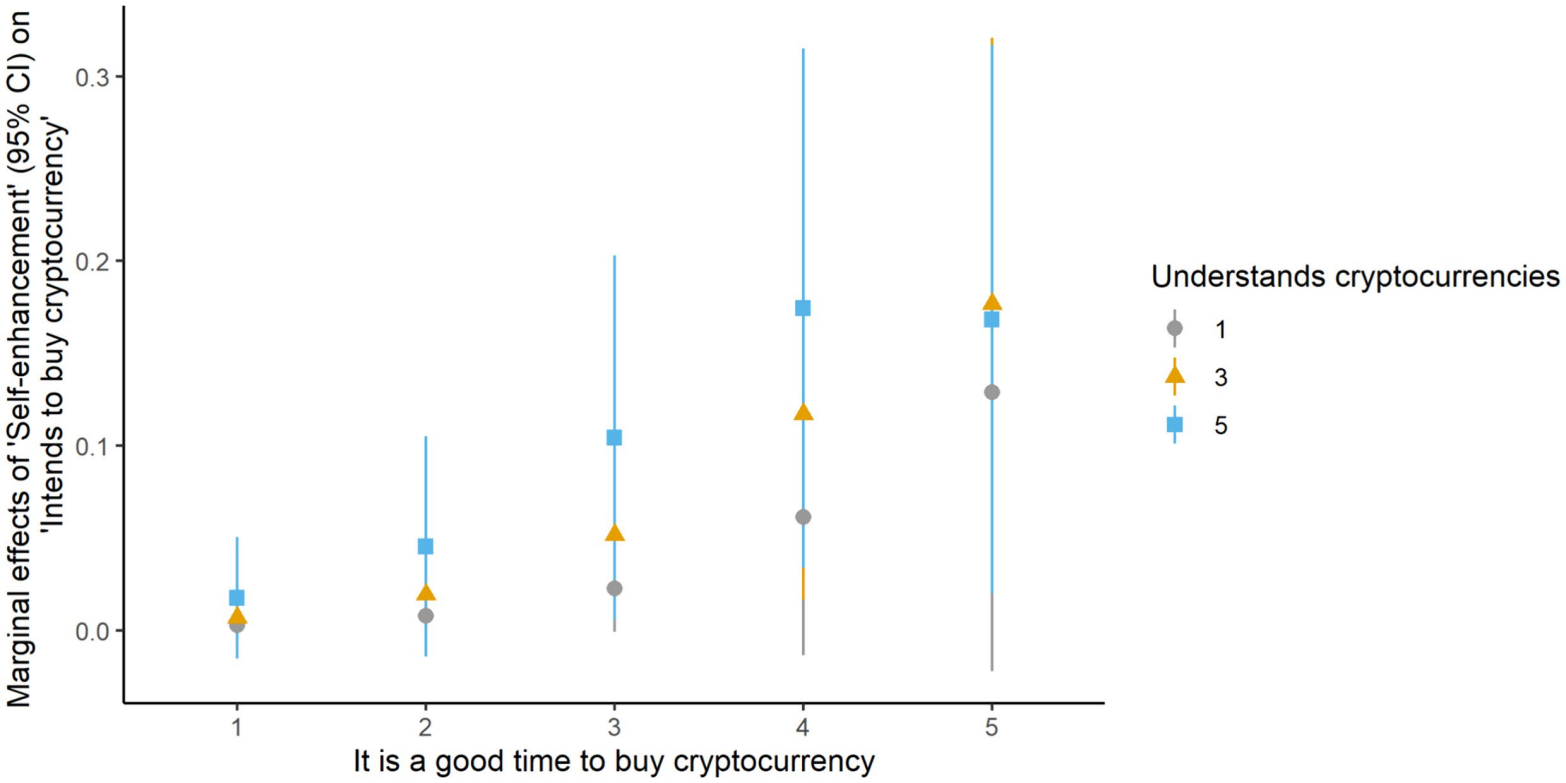
Marginal effects of self-enhancement on intention to buy cryptocurrency.

To gain clearer insights into hypotheses 3 and 5, we calculated and then plotted ME at levels of significant covariates, attitudinal beliefs, and perceived behavioral control. As shown in Figure 2, individuals who prioritized self-enhancement values were more likely to want to buy cryptocurrency in the future, especially if they believed it is a good time to buy cryptocurrency and had a good understanding of them.

**Figure 3:**
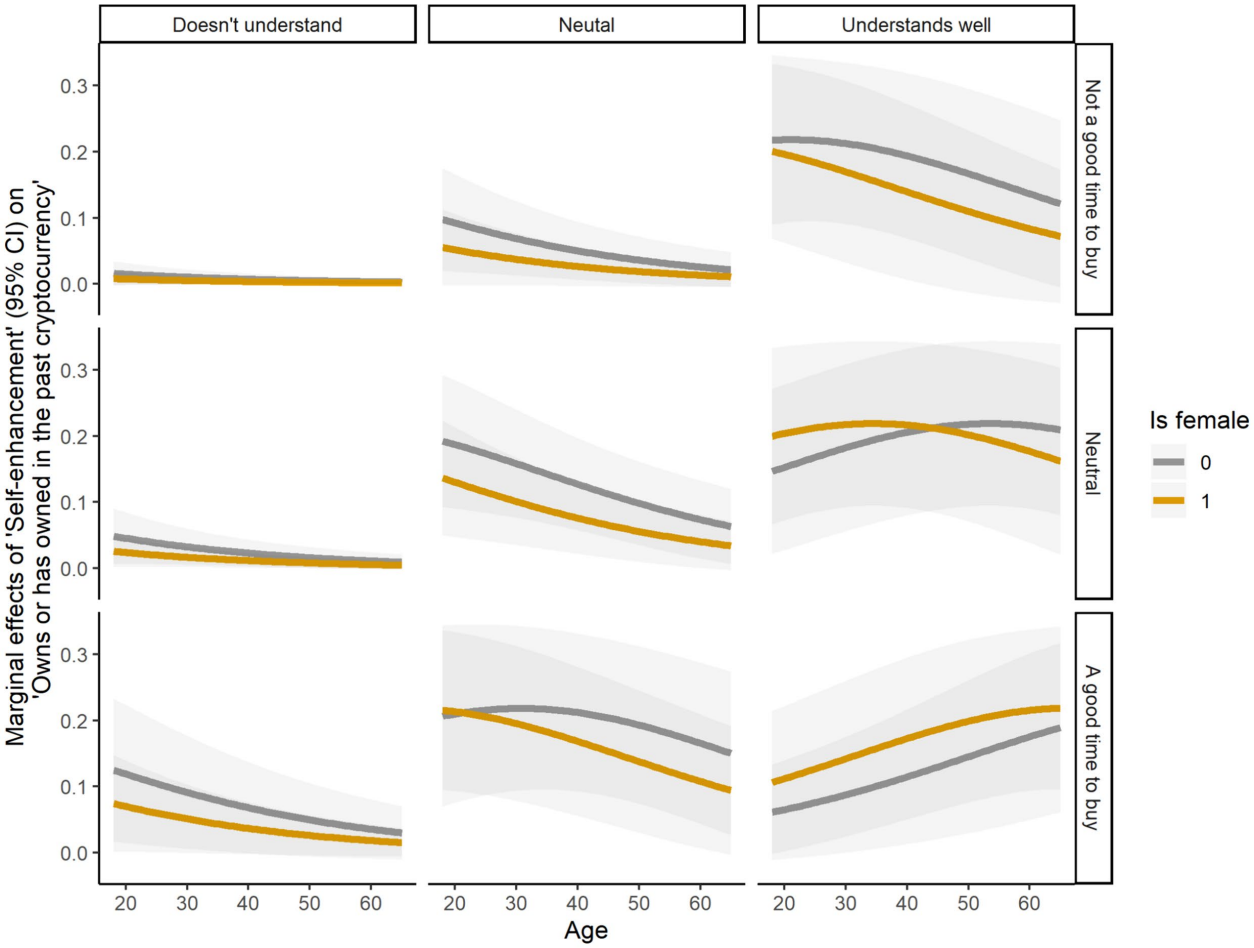
Marginal effects of self-enhancement on owns cryptocurrency, calculated for age, gender, understands cryptocurrency, and attitude about being a good time to buy cryptocurrency.

**Figure 4:**
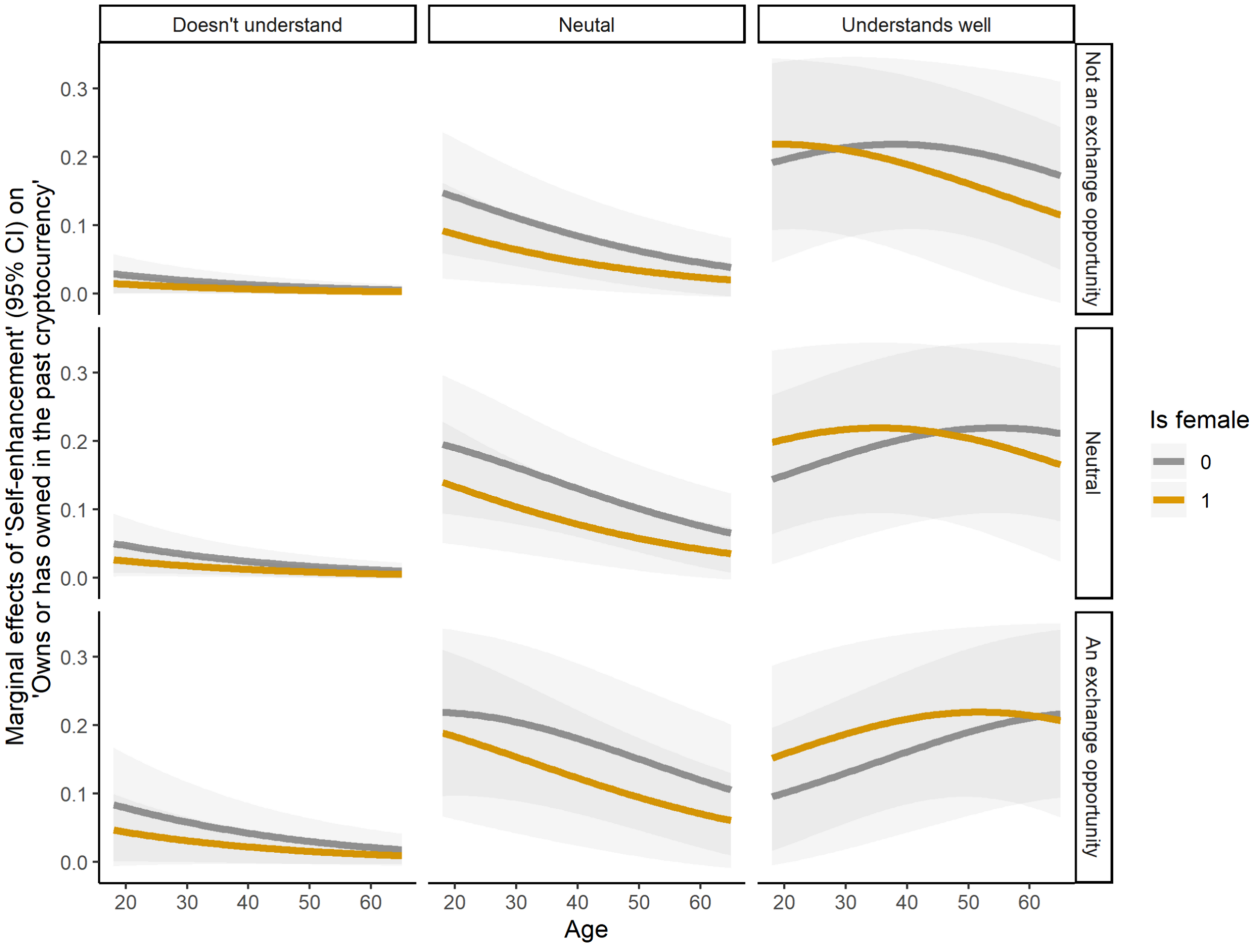
Marginal effects of self-enhancement on owns cryptocurrency, calculated for age, gender, understands cryptocurrency, and attitude about cryptocurrency being a money exchange opportunity.

Figures 3, 4 show a detailed picture of the ME of self-enhancement values on cryptocurrency ownership. The ME of self-enhancement values for women were almost always lower than for men, while the ME for younger participants tended to be higher than for older participants. Nonetheless, considering the significant effects of attitudinal beliefs–that it was a good time to buy cryptocurrency and that cryptocurrency can be easily exchanged for money–and of the perceived behavioral control–how well people understood cryptocurrency–the ME of self-enhancement were higher for women than for men, and for older than younger participants, among participants who understood well cryptocurrency and held positive attitudinal beliefs about cryptocurrency adoption. This indicates that prioritizing self-enhancement values generally increases the likelihood of owning cryptocurrency, especially for men and younger people. However, women and older individuals seem more likely to own cryptocurrency when they hold positive attitudes toward cryptocurrency adoption and have a better control over them.

## Discussion

Bitcoin, the first cryptocurrency to reach notoriety was introduced in 2008 with the mission to expediate a financial system independent from centralized financial institutions like banks or the State. Although digital currencies are currently being integrated in society, we still have limited psychological explanations for its fulminant evolution. The present research proposes that cryptocurrency adoption with the associated environmental, financial, and legal concerns reflect generalist existential needs of individuals that inform their values. Using the Theory of Planned Behavior as a guiding framework (TPB; Ajzen, 1991; Bosnjak et al., 2020), we hypothesized that values of openness-to-change (stimulation, self-direction) and self-enhancement (achievement, power) are related with cryptocurrency adoption at levels of awareness, intention to buy and ownership. Moreover, we predicted that attitudinal beliefs and a perceived behavioral control are likewise associated with the three levels of cryptocurrency adoption while we reasoned that they do not confound the effects of values but establish them further. Results of logit regressions generally provide favorable evidence for our hypotheses, whereas several observations deserve additional scrutiny.

We find that person-oriented values, which regulate how individuals express their personal interests and characteristics (Schwartz, 2012), play a role in cryptocurrency adoption. Although cryptocurrency appeals to those seeking stimulating activities and independent thinking (openness-to-change values) due to its novelty, it remains a complex monetary system that only those seeking personal success and deriving a sense of wellbeing from control over resources (self-enhancement values) do in fact adopt it. This finding instructs us that information concerning cryptocurrency may be instrumental. In other words, there might be a confirmation bias operating among the cryptocurrency adopters. Certain people may be curious or at least motivated to educate themselves about this new form of money but, it is specific knowledge associated with it that will eventually motivate them to (wanting to) own cryptocurrency, and one or more of the following topics may hold the key: environmental impact, potential for illegal activities, addictive gambling, and opportunity for financial gain.

Previous research has shown that male, young, highly educated, and financially privileged individuals are more likely to adopt cryptocurrencies (Sudzina et al., 2021; Steinmetz, 2022). Our findings support this notion while they also shed additional light on the nuances existing across levels of adoption. Although male and younger individuals are more likely to own cryptocurrencies, women and older individuals are better represented at levels of awareness and intention to buy, respectively. One possible explanation is that transitioning from an abstract ideal (value) to cryptocurrency ownership (behavior) unfolds as a part of decision-making processes typical for investors, where men typically enjoy more opportunities than women (e.g., Marinelli et al., 2017). Nonetheless, as our findings show, older women who value achievement and mastery over resources, are well-informed about cryptocurrency, and hold positive and practical beliefs about its financial benefits, may ultimately gain the upper hand over men. This scenario reminds us that women tend to be on average more averse to taking financial risks than men (Eckel and Grossman, 2008) and that pre-existent inequalities at the intersection of gender and finance can hinder women from building wealth (Wagner and Walstad, 2022).

One intriguing observation regarding cryptocurrency awareness was that individuals who value openness-to-change were more likely to have heard of cryptocurrency if they did not hold a university entrance degree (Abitur), with women having a higher likelihood than men overall. This contrasts previous results that higher educated individuals are more likely to own cryptocurrency (Steinmetz, 2022). Kromydas (2017) notes that the higher education system has shifted from its original mission of advocating for human development to promoting competition, which contrasts cryptocurrency’s mission of promoting individual financial independence from centralized monetary systems. Our findings suggest that the path toward formal higher education may not foster an interest in cryptocurrency among people who enjoy engaging in stimulating activities and independent thinking. However, with the available data we cannot rule out (family) financial situation as a confounding of educational attainment (Aakvik et al., 2019) and therefore it remains unclear whether higher education hampers cryptocurrency awareness or a precarious financial background promotes it among those who prioritize openness-to-change values.

Contrary to our expectations, we were not able to find an association between negative attitudinal beliefs and (lack of) cryptocurrency adoption. The present measures are informed by statements highlighting that cryptocurrency can facilitate illegal activities and that they are state regulated, which may have been insufficient in retrieving participants’ true negative attitudes toward digital currencies. It is possible that these attitudinal beliefs are not relevant in this value-attitude-behavior-hierarchy (see Milfont et al., 2010) operating at the individual level, albeit they might still play a role in explaining cryptocurrency adoption from a normative perspective (Stern et al., 1999)—for instance, whether participants favor efforts to implement cryptocurrency widely in society.

We explained 40% of cryptocurrency ownership and 15% of intention to buy it in the present data. This makes us confident that values together with attitudinal beliefs and a perceived behavioral control about digital currencies play a central role in explaining cryptocurrency ownership. Conversely, there seem to be other factors more potent in explaining the intention to buy. For instance, a derivative of the TPB applied in understanding the use of technology is the Technology Acceptance Model 3 (Venkatesh and Bala, 2008) which may inform a complementary framework in the study of intention to buy cryptocurrency (Jariyapan et al., 2021). Jariyapan et al. (2021) mention in their framework perceived risk, financial literacy, perceived usefulness and ease of use, as well as computer anxiety and computer self-efficacy, which is a welcome reminder that cryptocurrency combines technological novelty with financial elements.

### Limitations and future research

This study is exploratory and based on cross-sectional data collected in a quota sample of the German population through an online panel, which may limit the generalizability of our findings. Additionally, we were not able to examine causal relationships between basic human values and cryptocurrency adoption, so we must be cautious in our interpretations. Future research could seek to replicate our results with samples of students or by testing the value-cryptocurrency link in an experimental setting. A representative sample of the general population could also be examined to investigate the effects of values in cryptocurrency adoption at societal level. For instance, to understand why the typical investor’s profile is male, young, and better educated, when cryptocurrency’s initial proposal is precisely to make money more accessible to people in general.

The present research was carried out in a prototypical Western cultural context that in addition has a very high standard of living (Witte et al., 2020; UNDP, 2024)–Germany. A comparative analysis between cultural and socio-economic contexts is necessary to further uncover the links between human values and cryptocurrency adoption, especially concerning the question whether certain values have a universal effect, or the effects are contextually dependent. Values of people are known to differ cross-nationally contingent on several macro-level factors such as the GDPpc and life expectancy at birth (Witte et al., 2020). Thus, attempting to understanding the role of the socio-economic context in cryptocurrency adoption demands multidisciplinary integration, for instance, from economics, science and technology, and sociology (see for example Bhiamani et al., 2022).

Our study employed a dichotomous measure to evaluate cryptocurrency adoption. While this approach enabled us to predict whether individuals were aware of, intended to buy, or owned cryptocurrency, it did not allow us to examine the complexity and range of cryptocurrency adoption as influenced by basic human values. Additionally, the interpretations were made based on marginal effects derived from logit regressions which is not quite the same as testing for interaction effects directly in path models. We encourage researchers to use in the future multi-item scales (e.g., Jariyapan et al., 2021) that capture the different facets of the owning digital money, including concerns, purposes, and expectations.

One further limitation is that we operationalized basic human values at a high level of abstraction by using the higher order values proposed in the TBHV (e.g., Schwartz and Cieciuch, 2021). A more fine-grained operationalization of human values that considers specific motivational contents could provide deeper insights into the relationship between values and cryptocurrency adoption. For instance, longitudinal studies suggest that self-enhancement values change differently over time (Smallenbroek et al., 2023), with achievement reaching a plateau in adulthood and power declining thereafter. Future longitudinal studies can examine for example whether cryptocurrency adoption is less likely with declining power orientation, or more likely with a stable achievement orientation in people over time.

### Implications

The present findings come in the larger context of accelerating cryptocurrency regulation and rapid technological advances. A global study highlights core considerations in the regulatory sector (Xiong and Luo, 2024): (a) Whether cryptocurrency should be regulated in the first place, (b) whether existent financial frameworks should be adapted, or new ones developed entirely, (c) who the targets of these regulations should be, and (d) what are the current approaches to regulating cryptocurrency. Findings of Xiong and Luo (2024) show that most regulations are rather supportive with four countries including El Salvador that recognize cryptocurrencies as legal tender. Even payments of religious contributions such as the Zakat, Islam tax mechanism for the poor, are likewise becoming permissible through cryptocurrencies (Muneeza et al., 2022). Nonetheless, about 10 % of the countries at the global level adopt a full or partial ban (e.g., China, Republic of Moldova). Meanwhile, MiCA, the common regulatory framework of the European Union that was adopted in April 2023, emphasizes tracing of cryptocurrency trading similarly to traditional money transfers, highlights consumer protection, and safeguards against unlawful transactions. On this regulatory backdrop, central banks have in development digital currencies, for instance, the digital Euro (Brunnermeier and Landau, 2022). The race to cryptocurrency regulation is motivated by increasing adoption rates matched by increment fraudulent use.

Cryptocurrency is subject to the rapid technological advances. AI tools are being implemented in expediting trading (Kaur et al., 2024) in addition to being applied in the detection of fraud (Marasi and Ferretti, 2024). Meanwhile, security breaches in the blockchain technology, such as the infamous FTX collapse in November 2022 (Fu et al., 2023), motivate developers to explore novel techniques in ensuring security protocols while preserving anonymity (Rajamanickam and Chaturvedi, 2024). Among the explored techniques are the ring signature (encryption through a pool of participating signers) and the zero-knowledge proof (in-validation of transactions contingent of missing proof of transaction entirely). Moreover, developers have intensified their efforts lately in transitioning to more energy efficient security protocols such as from Proof-of-Work (PoW), native to Bitcoin, to Proof-of-Stake (PoS), which Ethereum implemented in 2022 with a proven whooping energy efficiency of 90%. A natural experiment of cryptocurrency investors’ portfolio before and after the “Merge” (the PoS adoption event by Ethereum) shows however a minimal shift toward the more climate friendly cryptocurrency (Baur and Karlsen, 2024, January 15) thus highlighting that the environmental impact caused by cryptocurrencies is not a top priority for current investors, although it might still be for future or undecided investors.

The trend in cryptocurrency adoption is upward, with the numbers attesting to that. Transparent regulations and technological updates that maintain anonymity while strengthening the security protocols might just contribute to an exponential cryptocurrency adoption in the end. However, just as not everyone has equal access to the internet or mobile phones (e.g., Marler, 2018; van Dijk, 2020)—two recent technologies that share similarities with cryptocurrency—, there are concerns that the adoption of cryptocurrency may be limited to a certain demographic, such as younger generations or those with more financial resources. Furthermore, the largely unregulated nature of cryptocurrency markets may enable some individuals to accumulate wealth at a faster rate than others, creating new forms of economic inequality. Meanwhile, the environmental impact caused by networks of computers sustaining the cryptocurrency systems is not a top priority among experienced investors.

Regulatory measures and policies are being developed to meet challenges surrounding cryptocurrency adoption. The present findings contribute to these efforts with the insight that human behavior including cryptocurrency ownership is at least partly motivated by value pursuit in people which underlie questions regarding self-needs and nature of relationship with others and the environment. We find that cryptocurrency ownership resonates with specific values in people (value motivational goals of power and achievement) which are often observed in certain demographics: educated younger men. Meanwhile, education and age correlate with more women being aware of cryptocurrencies if they also pursue value motivational goals of stimulating activities and independent thought. An actionable plan can be derived from the present research: Financial education for all can expediate the decline of gender (financial) inequality, and lessons can be learned from countries such as India and Brazil with a high dependency imbalance in disfavor of women (Bartholo, 2016; Jariwala and Dziegielewski, 2016). The development of financial autonomy through financial education workshops and governmental policies targeting the poor has been shown to improve financial behavior and financial management practices in women thus empowering them to become more autonomous individuals.

On the backdrop of this discussion, we recommend that policy makers and regulators recognize the importance of value pursuit in people as a fundamental behavioral motivator. Furthermore, we recommend policy makers and regulators to define and legislate cryptocurrencies in contexts of greater societal and environmental challenges that reflect existential questions in people from which they derive motivational goal pursuit. Thus, we propose that cryptocurrencies may have started as an alternative financial system, but it has since evolved to encompass other domains of life. This evolution is currently insufficiently accommodated in existent regulations and policies that focus primarily on the economic dimension.

## Conclusion

The present findings suggest that cryptocurrency currently align with certain motivational goals of people, and that adoption at the individual level can be predicted in the Theory of Planned Behavior. These insights may be useful for policy makers and researchers to consider mitigating the risk of cryptocurrency creating new forms of inequality.

## Data availability statement

Data, scripts and subsequent study materials are open access via: https://osf.io/n7qw4/.

## Ethics statement

Ethical review and approval was not required for the study on human participants in accordance with the local legislation and institutional requirements. Written informed consent to participate in this study was provided by the participants.

## Author contributions

AS: Conceptualization, Data curation, Formal analysis, Funding acquisition, Investigation, Methodology, Project administration, Resources, Software, Supervision, Validation, Visualization, Writing – original draft, Writing – review & editing. MP: Conceptualization, Writing – original draft, Writing – review & editing. CL: Conceptualization, Funding acquisition, Methodology, Writing – original draft, Writing – review & editing.

## Footnotes

^1^https://www.gesis.org/en/allbus/overview
