# Supplementary material for: Basic human values and the adoption of cryptocurrency
Source: Front Psychol. 2024 Aug 16;15:1395674. doi: 10.3389/fpsyg.2024.1395674 (PMC11362991; doi:10.3389/fpsyg.2024.1395674)
Supplement: Supplementary file 1 [file Table_1.DOCX]

Basic Human Values and the Adoption of Cryptocurrency

Supplement Material


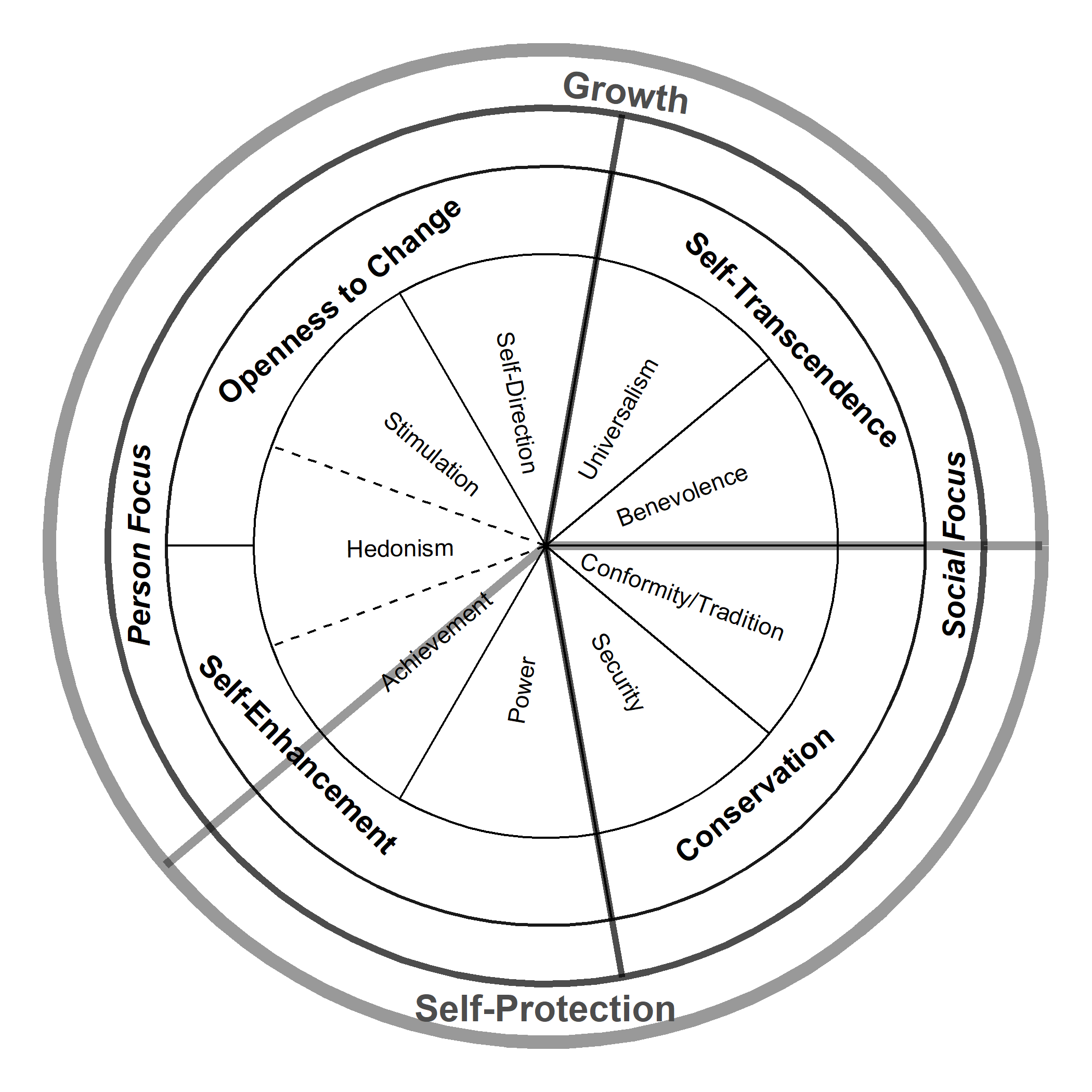


**Figure 1.**

*Human values in The Theory of Basic Human Values.*


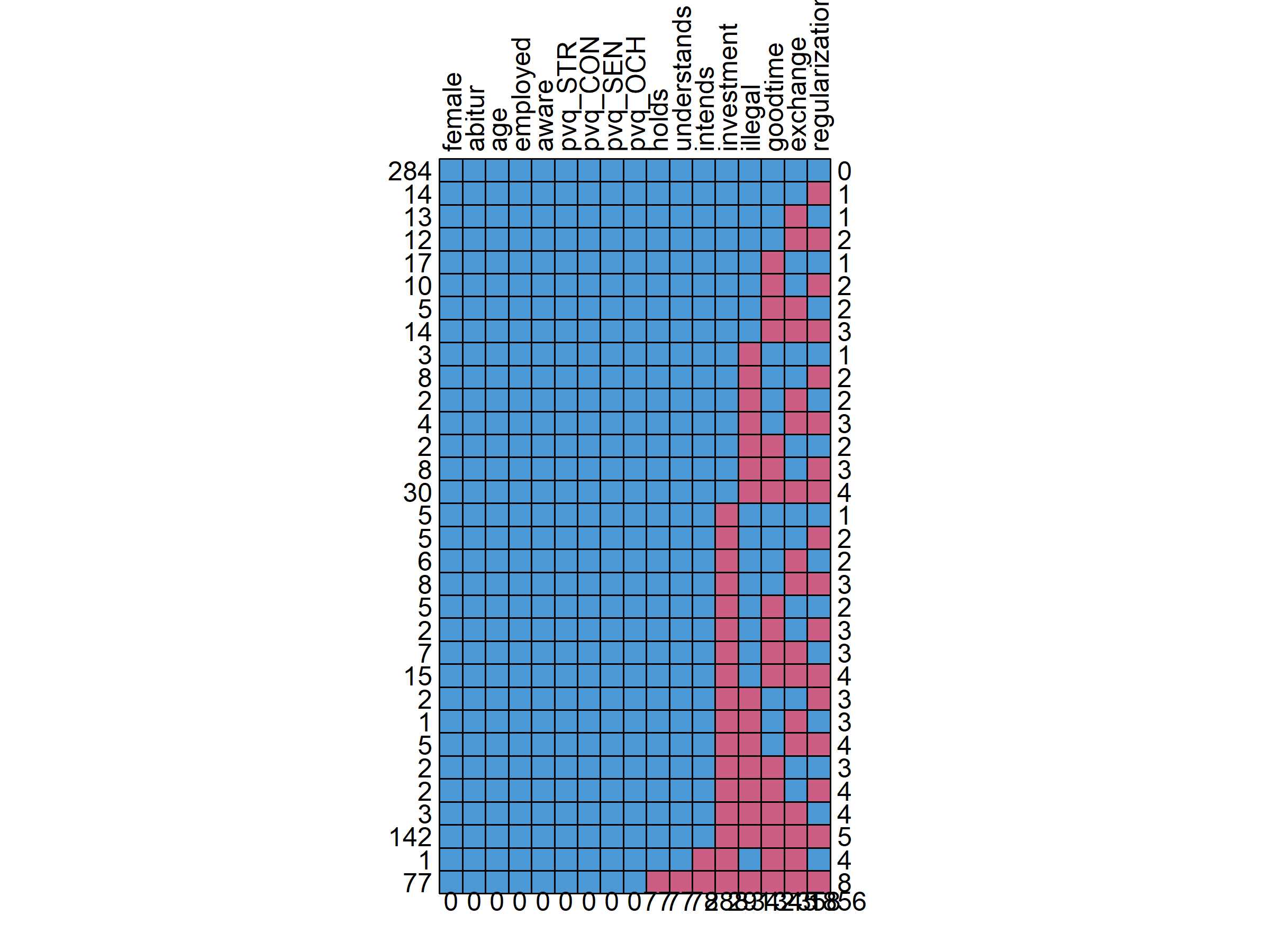


**Figure 2.**

*Data Missing Pattern.*


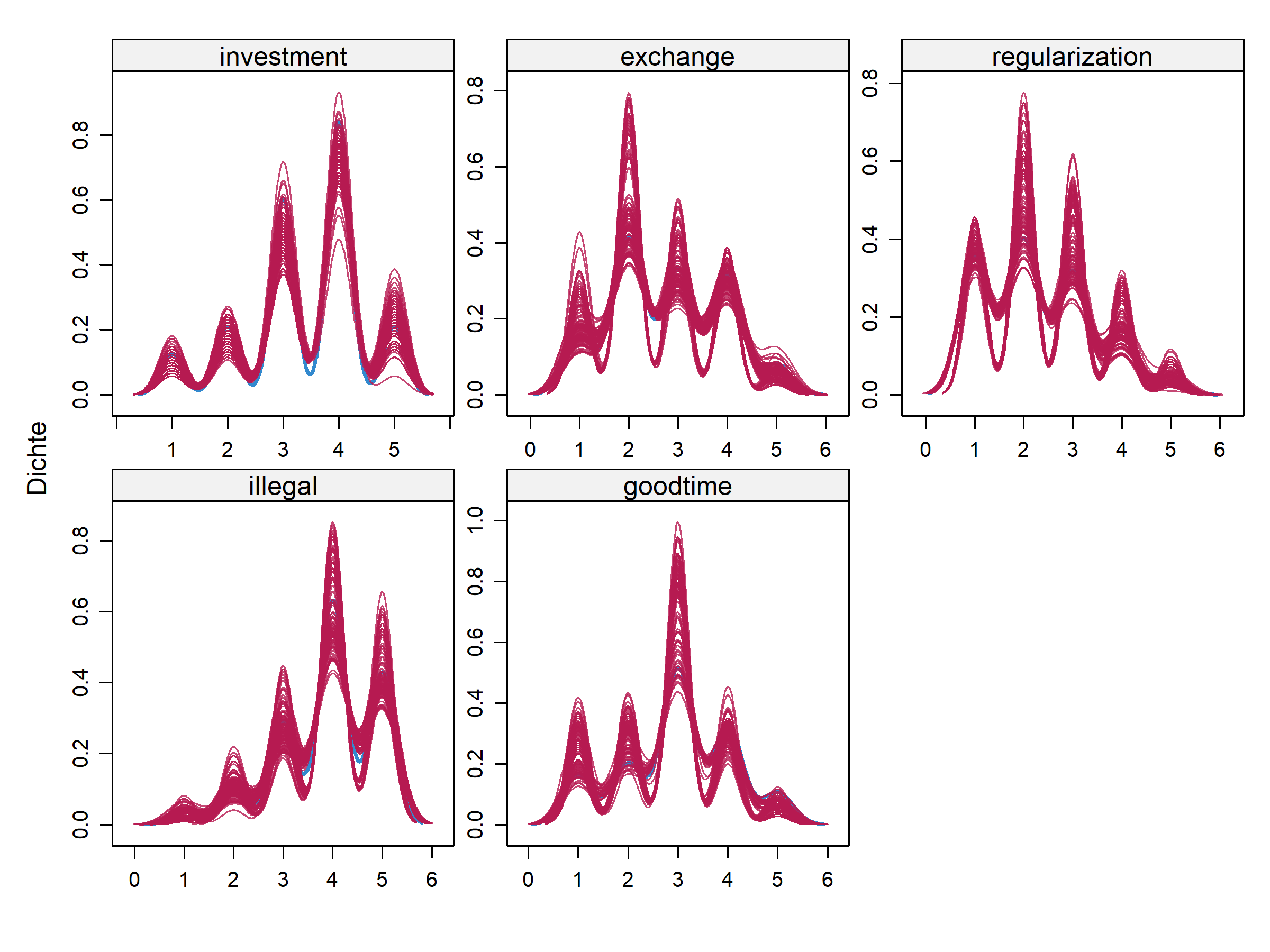


**Figure 3.**

*Density Plots for Imputed Data on the Attitudinal Indicators.*


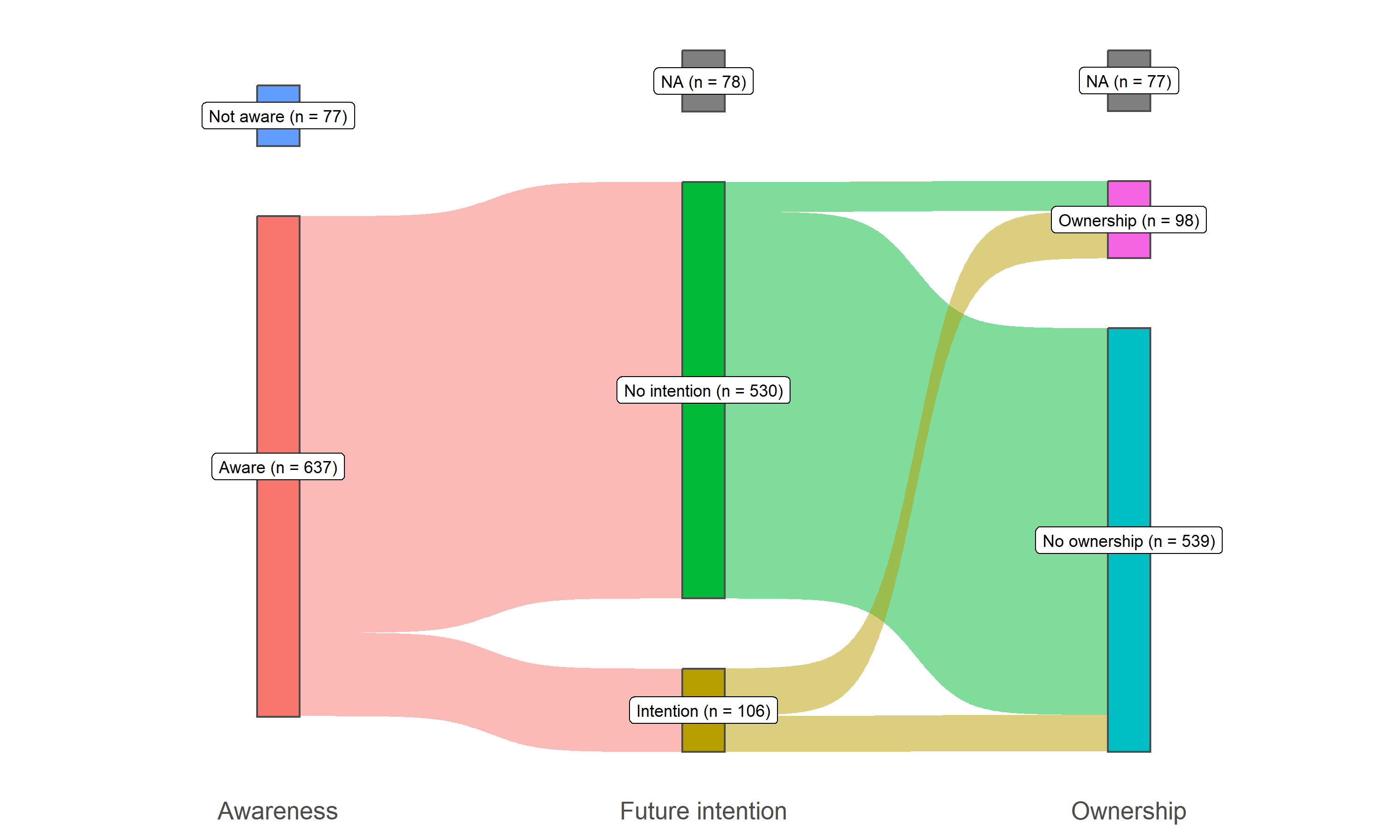


**Figure 4.**

*Distribution of Cryptocurrency Adoption in The Sample at Levels of Awareness, Intention to Buy and Ownership.*
